# Development of a novel *in vitro* model to study the modulatory role of the respiratory complex I in macrophage effector functions

**DOI:** 10.1371/journal.pone.0291442

**Published:** 2023-09-19

**Authors:** Pablo Serrano-Lorenzo, Dino Gobelli, Rocío Garrido-Moraga, María J. Esteban-Amo, José R. López-López, Antonio Orduña, Miguel A. de la Fuente, Miguel A. Martín, María Simarro

**Affiliations:** 1 Hospital 12 de Octubre Research Institute (imas12), Madrid, Spain; 2 Biomedical Network Research Centre on Rare Diseases (CIBERER), Instituto de Salud Carlos III, Madrid, Spain; 3 Mitochondrial Disorders Laboratory, Clinical Biochemistry Department, Hospital Universitario 12 de Octubre, Madrid, Spain; 4 Department of Cell Biology, Histology and Pharmacology, Faculty of Medicine, University of Valladolid, Valladolid, Spain; 5 Unit of Excellence Institute of Biomedicine and Molecular Genetics (IBGM), University of Valladolid and Spanish National Research Council (CSIC), Valladolid, Spain; 6 Department of Department of Biochemistry and Molecular Biology and Physiology, Faculty of Medicine, University of Valladolid, Valladolid, Spain; 7 Division of Microbiology, Hospital Clínico of Valladolid, Valladolid, Spain; 8 Department of Microbiology, University of Valladolid, Valladolid, Spain; Augusta University, UNITED STATES

## Abstract

Increasing evidence demonstrate that the electron transfer chain plays a critical role in controlling the effector functions of macrophages. In this work, we have generated a *Ndufs4*−/− murine macrophage cell lines. The *Ndufs4* gene, which encodes a supernumerary subunit of complex I, is a mutational hotspot in Leigh syndrome patients. *Ndufs4*−/− macrophages showed decreased complex I activity, altered complex I assembly, and lower levels of maximal respiration and ATP production. These mitochondrial respiration alterations were associated with a shift towards a pro-inflammatory cytokine profile after lipopolysaccharide challenge and improved ability to phagocytose Gram-negative bacteria.

## Introduction

Mammalian NADH:ubiquinone reductase or complex I (CI) is the largest enzyme of the electron transport chain (ETC) and consists of 45 subunits, 38 encoded by nuclear genome and 7 subunits by mitochondrial genome [1]. It couples electron transfer from NADH to ubiquinone with transmembrane proton pumping contributing to the proton motive force used for ATP synthesis. The catalytic core of CI comprises 14 evolutionary conserved proteins which are encoded by the nuclear NDUFV1, NDUFV2, NDUFS1, NDUFS2, NDUFS3, NDUFS7, and NDUFS8 genes and the mitochondrial ND1, ND2, ND3, ND4, ND4L, ND5 and ND6 genes [1]. In addition to these core subunits, mammalian CI also contains 31 nuclear encoded supernumerary subunits which are believed not to be involved in the catalytic reaction but are necessary for the correct assembly and functioning of the CI [2]. Defects in human CI is the most frequently encountered ETC enzyme deficiency and result in characteristic pathologies affecting organs with high energy demand, such as muscle and brain [3].

NADH Dehydrogenase (Ubiquinone) Fe-S protein 4 (NDUFS4) is one of the supernumerary subunits of CI and, importantly, the gene encoding NDUFS4 is a hot spot for pathogenic mutations that mostly cause Leigh or Leigh-like syndromes. These syndromes are early onset and often fatal neurological disorders clinically characterized by motor and intellectual retardation, seizures, and respiratory insufficiency [4, 5]. Similarly, global and neuron/glia specific *Ndufs4*−/− mice showed retarded growth, loss of motor ability, breathing abnormalities, and died within ~ 50 days after birth [6, 7]. All the tissues examined (pancreas, kidney, liver, lung, brain, heart, and muscle) from global *Ndufs4*−/− mice exhibited a significant decrease in CI activity when compared to the control values, in addition to the absence of fully assembled CI [8].

In addition to supporting energy demands, increasing evidence demonstrate that ETC plays a critical role in controlling the activation and functions of certain immune cells, especially in macrophages [9]. In this work, we further investigate the role of ETC in the effector functions of macrophages through the generation and characterization of CRISPR/Cas9-mediated *Ndufs4*−/− RAW 264.7 cells: a murine macrophage cell line.

## Materials and methods

### Cell lines

The murine macrophage RAW 264.7 cell line was obtained from the American Type Culture Collection (TIB-71). *Ndufs4*−/− RAW 264.7 was generated using CRISPR/Cas9 tool as described in the subsequent subsections.

### Plasmids construction

The targeting vector was designed to delete *Ndufs4* using Cas9-induced homology-independent targeted integration (HITI) strategy [10]. gRNA sequences against the exon 1 of *Ndufs4* was designed using CRISPOR tool [11]. Forward and reverse oligonucleotides containing the guide sequences for *Ndufs4* (CL1 and CL2) were annealed and cloned into the BbsI site of pX330-U6-Chimeric_BB-CBh-hSpCas9 (#42230, Addgene) [12] generating a plasmid named pX330-Ndufs4. For the HITI donor plasmid production, forward and reverse primers were designed to contain the gRNA sequences for *Ndufs4* (CL3 and CL4), the PAM sequence, stop codons (CL3) and 21–22 nucleotides corresponding to the 5’ (CL3) and 3’ (CL4) flanking regions of the floxed blasticidin cassette of the pBS-Blast plasmid. PCR products were cloned into BamHI and SalI sites of pBluescript II KS+ (#212207, Stratagene). pBS-Blast plasmid was previously generated in our laboratory, and it is a pBluescript II based plasmid containing a blasticidin resistance cassette flanked by loxP sequences. The resultant donor plasmid was named pBLAST-Ndufs4. The sequences of the cloning primers CL1-4 are shown in S1 Table.

### Generation of *Ndufs4*−/− macrophages

RAW 264.7 cells were transfected with pX330-Ndufs4 plasmid and the HITI donor plasmids pBLAST-Ndufs4 at a molar ratio of 3:1 and selected in medium containing 2 μ/ml blasticidin. Correctly targeted clones were identified by Sanger sequencing of PCR products. The absence of Ndufs4 protein was confirmed by Western blotting.

### Mitochondrial respiratory chain (MRC) activities

Activities of the respiratory chain complexes in cells were measured spectrophotometrically using the method of Bujan et al. [13]. Two 175 cm2 flasks of confluent cells were collected by centrifugation at 800 x g for 5 min. Pellets were resuspended in 300 μL homogenization buffer (mannitol buffer). The mannitol buffer pH 7.2 consisted of 225 mM D-mannitol, 75 mM sucrose, 10 mM Tris-HCl and 0.1 mM EDTA. The cell suspension was sonicated twice for 5s at 200 watts in an ice bath. Cell homogenates were maintained in the ice bath prior to the spectrophotometric enzyme assays.

### Mitochondrial respiration assays

Oxygen consumption rate (OCR) was assessed in the extracellular analyzer XFp (Seahorse Agilent Technologies). Mitochondrial respiration assays were performed following the described protocol [14] with minor modifications in reagents concentrations: 2.6 μM oligomycin (#75351, Sigma-Aldrich), 1.0 μM carbonyl cyanide 4-(trifluoromethoxy)-phenyl-hydrazone (FCCP) (C2920, Sigma-Aldrich) and 1.0 μM rotenone/antimycin A (R8875 and A8674, respectively, both from Sigma-Aldrich). 40.000 cells/well were seeded for approximately 24 hours on XFp plates prior to performing the test. Data was obtained using Agilent Seahorse Wave 2.6.3.5 software (Seahorse Agilent Technologies). When indicated, cells were preincubated during 4 hours with 200 ng/mL Lipopolysaccharides (LPS) from Escherichia coli O55:B5 (L6529, Sigma-Aldrich) before testing.

### Measurement of mitochondrial membrane potential (MMP), mitochondrial reactive oxygen species (mitoROS) and NAD+/NADH ratio

MitoTracker Green (for total mitochondrial mass), MitoTracker Red CMXRos (for MMP) and MitoSOX (for mitoROS) staining were performed according to manufacturer’s instructions. For flow cytometry analysis, data were acquired with a Gallios Flow Cytometer and analyzed with Kaluza version 1.1. The NAD+/NADH Quantification Kit (Sigma Aldrich, St. Louis, USA) was used to measure the NAD+/NADH ratio according to the manufacturer’s instructions. Briefly, a pellet of 3x10^6^ cells was collected, deproteinized using a 10 kDa molecular weight cut-off, and the NAD+/NADH ratio was measured through colorimetric detection at 450nm. To detect only NADH, NAD+ was decomposed by maintaining it at a temperature of 60°C for 30 minutes.

### Purification of mitochondria-enriched protein fractions

Cell pellets were resuspended in 4 ml in isotonic buffer containing 250 mM Sucrose, 20 mM Tris-HCl pH 7.8, 0.2 mM EDTA and 0.1 mM phenylmethylsulfonyl fluoride (sucrose-PMSF buffer). Subsequently, sample homogenization was carried out in ice and broken cells were first centrifuged at 800 x g for 10 minutes. Next, the supernatant containing cytoplasm, membrane and mitochondria was centrifuged again at 20,000 x g for 10 minutes. Then the pellets were resuspended (mitochondria enriched fraction) in sucrose-PMSF buffer and centrifuged again at 20,000 x g for 10 minutes. The mitochondrial pellet was resuspended in an appropriate volume (~200–500 μl) of a sucrose buffer containing 10mM HEPES pH 7.6 and 0.5 M sucrose. Protein concentration was determined using the Pierce MicroBCA protein assay kit (#23235, Thermo Fisher Scientific).

### Sodium dodecyl-sulfate polyacrylamide gel electrophoresis (SDS-PAGE)

Total cell lysates were prepared using RIPA buffer and enriched mitochondrial lysates were obtained as described in 2.6. 30–50 μg of protein from total cell and mitochondrial lysates were separated on SDS-PAGE gels and transferred to a polyvinylidene difluoride (PVDF) membrane, 0.45 μm pore size (Thermo Fisher Scientific) by conventional procedures.

### Blue native polyacrylamide gel electrophoresis (BN-PAGE)

Mitochondrial pellets were resuspended in 100 to 200 μl buffer containing 1.5 M aminocaproic acid and 50 mM Bis-Tris (pH 7.0). Samples were solubilized using digitonin at a detergent-to-protein ratio of 4:1 and incubated on ice for 15 min. After centrifugation for 30 min at 20,000 x g at 4°C, the supernatant was combined with 10 to 20 μl of sample buffer (750 mM aminocaproic acid, 50mM Bis-Tris, 0.5 mM EDTA, 5% Coomassie Brilliant Blue G-250) prior to loading. Invitrogen NativePAGE 3–12% Bis-Tris mini protein gels (Thermo Fisher Scientific) were loaded with 30–40 μg of mitochondrial protein and processed for BN-PAGE as previously described [15]. Finally, proteins were transferred to PVDF membranes.

### Western blotting

After electroblotting the proteins onto PVDF membranes, they were detected by incubating the membranes with the following antibodies: anti-ATP5A (ab14748, Abcam), anti-ATP5B (ab128743, Abcam), anti-β-actin (A3854, Sigma-Aldrich), anti-COX5A (ab110262, Abcam), anti-NDUFA9 (ab14713, Abcam), anti-NDUFS4 (PA5-92940, Thermo Fisher Scientific), anti-NDUFV1 (sc-100566, Santa Cruz Biotechnology), anti-SDHA (ab14715, Abcam), and anti-UQCR2 (ab14745, Abcam). Immunoreactive bands were detected with an Amersham ECL Prime Reagent (GE- Healthcare) in a ChemiDoc MP Imager (Bio-Rad).

### CI in-gel activity assay (IGA)

A Blue Native gel was used for CI IGA. Gel was incubated at room temperature with a 2mM Tris-HCl pH 7.4 solution, 0.1 mg/mL NADH (as a substrate), and 2.5 mg/mL nitro blue tetrazolium (NBT, as the electron acceptor). NBT is reduced to formazan by CI and forms purple precipitates at the site of CI-containing respiratory supercomplexes (SCs). The displayed image was captured after 180 min of incubation in the IGA solution.

### Measurement of cytokines

Macrophages were stimulated with 100 ng/ml LPS, and cells were collected at 4 hours for cytokines mRNA quantification by real-time PCR and supernatants were collected at 8 hours for cytokines measurements by ELISA. In all the experiments, macrophages (3x10^5^) were seeded in 24 well plate for approximately 24 hours prior to LPS stimulation. Total RNA was extracted using TRIzol reagent (Thermo Fisher Scientific). RNA (1 μg) was reverse transcribed using First Strand cDNA Synthesis Kit (K1612, Thermo Fisher Scientific) to generate cDNA that was quantified by real-time PCR analysis with the LightCycler 480 Instrument (Roche Diagnostics) using PowerUp SYBR Green PCR Master Mix (A25742, Thermo Fisher Scientific). Primers pairs were designed for amplification of Hprt (RT1/RT2), IL-1β (RT3/RT4), IL-6 (RT5/RT6), IL-10 (RT7/RT8), and Tnf-α (RT9/RT10). Primer sequences are provided in S1 Table. Relative mRNA expression was obtained using the ΔΔCt method using Hprt as reference gene. ELISA kits from Peprotech were used for the detection of IL-1β (#900-M47), IL-6 (#900-M50), IL-10 (#900-M53) and TNF-α (#900-M54). Absorbance was measured on a VersaMax microplate reader (Molecular Devices).

### Phagocytosis assays

Phagocytosis of fluorescently labeled bacteria was measured by flow cytometry. Heat-killed *Escherichia coli* (80°C, 15 min) were incubated with a 0.1 mg/ml solution of Fluorescein-5-isothiocyanate (FITC) at 37°C for 30 min and washed three times with PBS prior to use. FITC-labeled bacteria were added to 5x10^5^ macrophages at a 100:1 ratio for 30 min at 37°C. Macrophages were then washed three times with cold PBS, and extracellular FITC-labeled bacteria were quenched with a 60 s wash in trypan blue (0.2 mg/ml). Macrophages were fixed with 4% paraformaldehyde and analyzed using a Gallios Flow Cytometer (Beckman Coulter Life Sciences). The bacterial strain used in these assays was *Escherichia coli* DH5α (#18258012, Thermo Fisher Scientific).

### Statistical analysis

Statistical analyses were performed using GraphPad Prism software. Unpaired Student’s t-test (two-tailed) or Mann-Whitney U test (two-tailed) were used when comparing two groups. test. Values of p < 0.05 were considered statistically significant. In the figures, each point represents a biological replicate and, if no other indication is made, data are expressed as the mean ± SEM.

## Results

### Generation of NDUFS4-deficient macrophage cell lines

**Fig 1:**
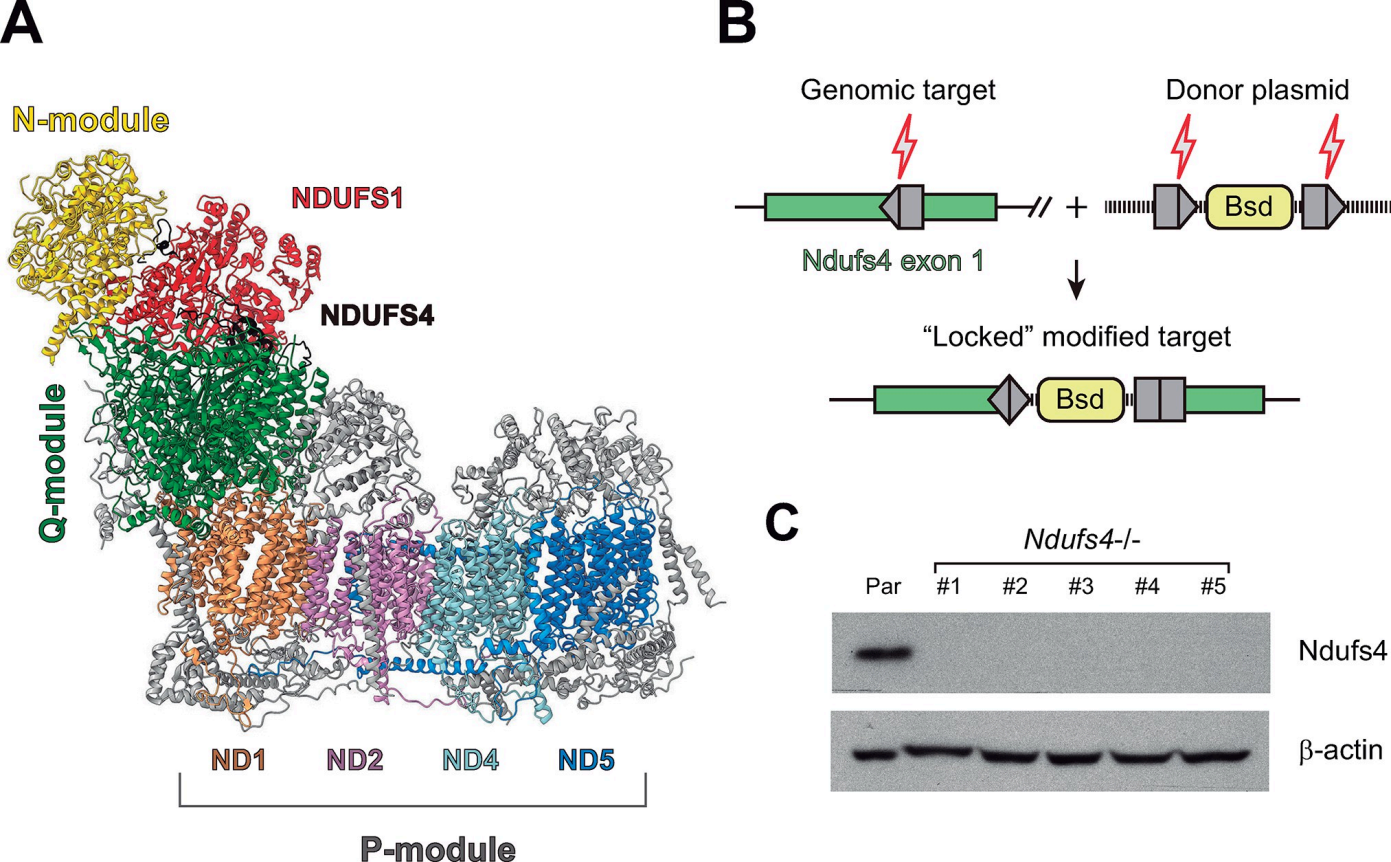
Generation of *Ndufs4−/−* macrophage cell lines. (A) Structural model for ovine (*Ovis aries*) mitochondrial complex I (CI, Ref: 5LNK) [17] displaying the location of the NDUFS4 subunit (colored in black). UCSF ChimeraX (v1.5) [18] was used for visualization. (B) General Cas9 HITI strategy to ablate Ndufs4. Cas9 produces a double stranded DNA break at specific target sequences within the first exon of *Ndufs4*. Cas9 also excises the HITI donor by cleaving the same target sequence flanking the blasticidin cassette to be inserted. The excised HITI donor is ligated into the genomic site through the NHEJ pathway. The forward integration depicted in the scheme is “locked” and cannot be processed further. A reverse integration could be corrected by continuous excision/repair cycles in virtue of flanking target sites reconstitution. Grey pentagons represent Cas9/gRNA target sequences. Black lines within pentagons indicate Cas9 cleavage sites. (C) Whole cell lysates from each resultant knockout cell line were analyzed by Western blotting using antibodies against Ndufs4 or β-actin (loading control). Correctly targeted clones were named *Ndufs4−/−* followed by a serial number; Par, parental cell line.

The CI has an L-shaped structure with one arm protruding into the matrix and the other arm embedded in the inner membrane (Fig 1A). The distal half of the matrix arm forms the N module, which includes core subunits NDUFS1, NDUFV1, NDUFV2, and is responsible for the oxidation of NADH into NAD+. The Q module, which represents the proximal half of the peripheral arm, contains core subunits NDUFS2, NDUFS3, NDUFS7, NDUFS8, and delivers electrons to ubiquinone via eight iron-sulfur clusters. The membrane arm (P-module) includes all the mtDNA-encoded subunits (core subunits ND1 to ND6 and ND4L) and is responsible for proton translocation (P-module). Ndufs4 (identified in black in Fig 1A) is a small supernumerary subunit of 18 kDa. It is incorporated into the complex during the later stages of the assembly process [16] and, as stated in the introduction, is required for proper CI activity and assembly in various tissues [8]. Here, we aimed to investigate the impact of the absence of Ndufs4 on mitochondrial and effector functions in macrophages. To this aim, we constructed a cell model of Ndufs4 gene knockout in murine macrophage cell line RAW264.7 using HITI strategy as described in Materials and Methods and depicted in Fig 1B. A guide RNA (gRNA) was designed targeting a sequence within exon 1 located ~30 bp downstream the ATG start codon. Five *Ndufs4*−/− clones (#1 to #5) were obtained that showed a complete absence of Ndufs4 expression compared to the parental cell line (Fig 1C).

### The absence of NDUFS4 impairs mitochondrial respiration and prevents respirasome formation

**Fig 2:**
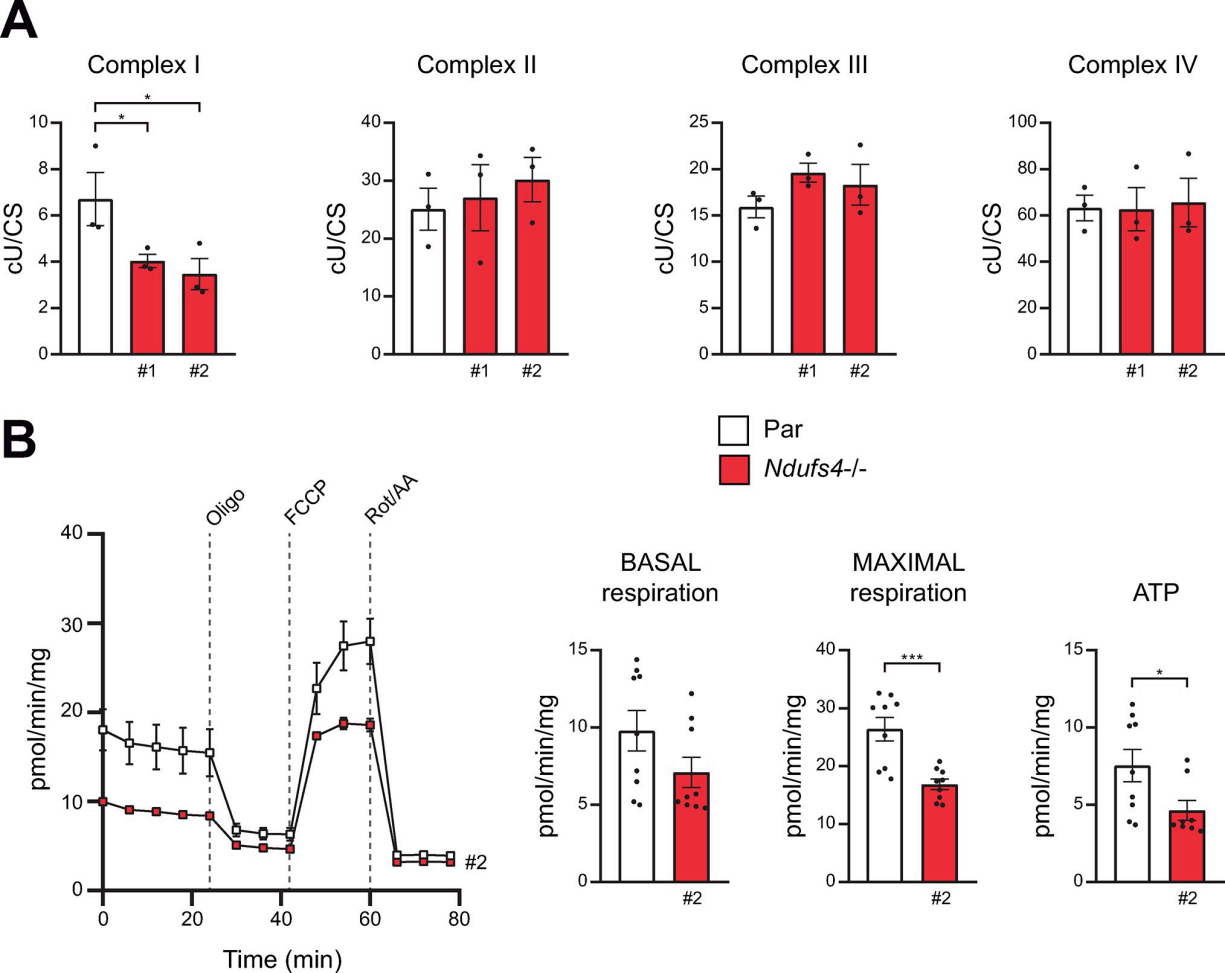
Respiration in *Ndufs4−/−* macrophages. (A) Activities of MRC complex (I–IV) were assayed spectrophotometrically, and the results were normalized to citrate synthase (CS) activity in mitochondria isolated from parental (Par) and *Ndufs4−/−* RAW 264.7 cells. (B) Left panel, a representative experiment showing OCR in RAW 264.7 sublines before and after the sequential addition of oligomycin (2.6 μM), FCCP (1 μM), and a combination of rotenone (Rot) and antimycin A (AA) (1 μM). Right panel, basal respiration, maximal respiration, and ATP production. Ns, not significant; *, P <0.05; **, P <0.01; ***, P<0.005; ****, P<0.001. Each point represents a biological replicate. Data are shown as the mean ± SEM.

In order to study the impact of the absence of NDUFS4 on mitochondrial respiratory functions, we first measured spectrophotometrically the MRC activities in mitochondria-enriched preparations of *Ndufs4*−/− cells. Subsequently, we normalized the complexes activities to respective citrate synthase activities (Fig 2A). As expected, *Ndufs4*−/− cells showed a significantly lower level in CI activity relative to control cells (56% of control values). No significant differences were found in the activities of the MRC II-IV complexes between *Ndufs4*−/− and control cell lines (Fig 2A). As expected, the reduction of CI activity in *Ndufs4*−/− cells, when the CI activity was normalized to the activities of CII and CIV (S1 Fig), was similar to that observed when the CI activity was normalized to CS (Fig 2A). We next assessed the mitochondrial respiratory profile of *Ndufs4*−/− macrophages. Oxygen consumption rate (OCR) was measured before and after the sequential addition of oligomycin (ATP synthase inhibitor), FCCP (H+ ionophore), and rotenone (CI inhibitor) (Fig 2B). *Ndufs4*−/− macrophages showed lower levels of basal respiration, and significantly lower levels of maximal respiration and ATP production than control cells, revealing a global decay in the electron flux through the MRC (Fig 2B). Additionally, *Ndufs4*−/− cells had increased basal levels of mitoROS without any significant decrease in their MMP (S2 Fig). Intriguingly, despite these alterations in mitochondrial function, *Ndufs4*−/− macrophages proliferated normally and displayed no obvious apoptotic signs (S3 Fig).

Since NDUFS4 plays a crucial role in the binding between the N-module and the rest of the enzyme [8], we explored whether the protein levels of two CI subunits close to NDUFS4, NDUFV1 (N-module) and NDUFA9 (Q-module), were altered in *Ndufs4*−/− macrophages. As shown in S4 Fig, the levels of NDUFV1 and NDUFA9 in *Ndufs4*−/− macrophages were similar to those observed in parental cells, indicating that the absence of NDUFS4 did not alter their stability.

**Fig 3:**
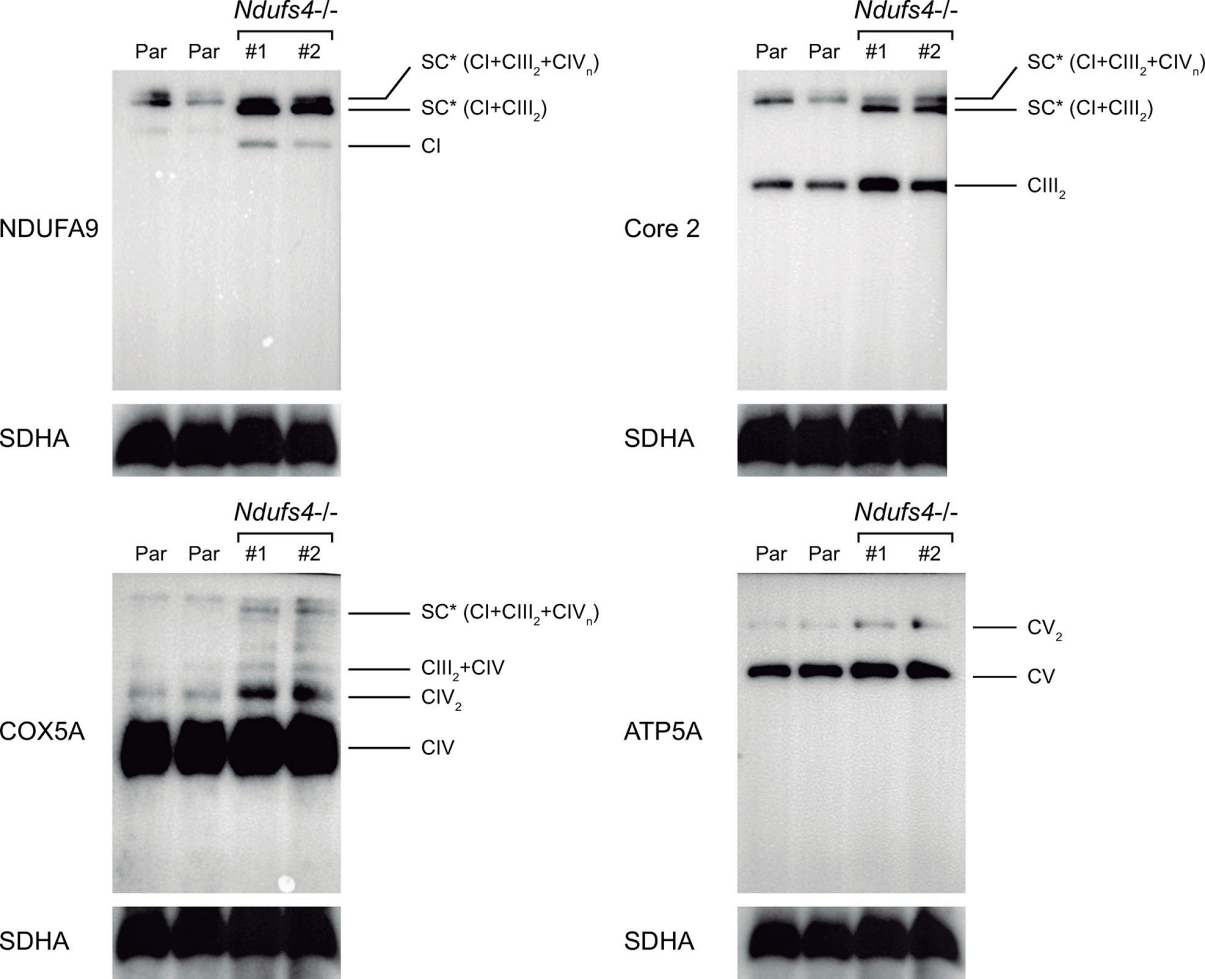
Respirasome assembly in *Ndufs4−/−* macrophages. 1D-BNE showing OXPHOS complexes I to IV (CI-CIV) and SCs. Western blot analysis was performed using antibodies against CI (NDUFA9), CIII (Core 2), CIV (COX5A), and CV (ATP5A). Asterisks (*) indicate lower molecular weight SC. Loading control, CII subunit SDHA.

Next, we analyzed the distribution pattern of free MRC complexes and SCs by 1D BN-PAGE followed by western blot analyses using antibodies against NDUFA9 (CI), core 2 (CIII), COX5a (CIV) and ATP5A (CV). We observed *Ndufs4*−/− cell lines lacked fully assembled free CI and CI-containing SCs (SC I + III_2_ + IVn, SC I + III_2_). Instead, we noted a significant accumulation of lower molecular weight SCs, suggesting the presence of partially assembled SCs (Fig 3). Higher levels of CIII_2_ and CIV_2_ were also observed compared to control cells. No significant differences were found either in the monomer or dimer levels of CV.

**Fig 4:**
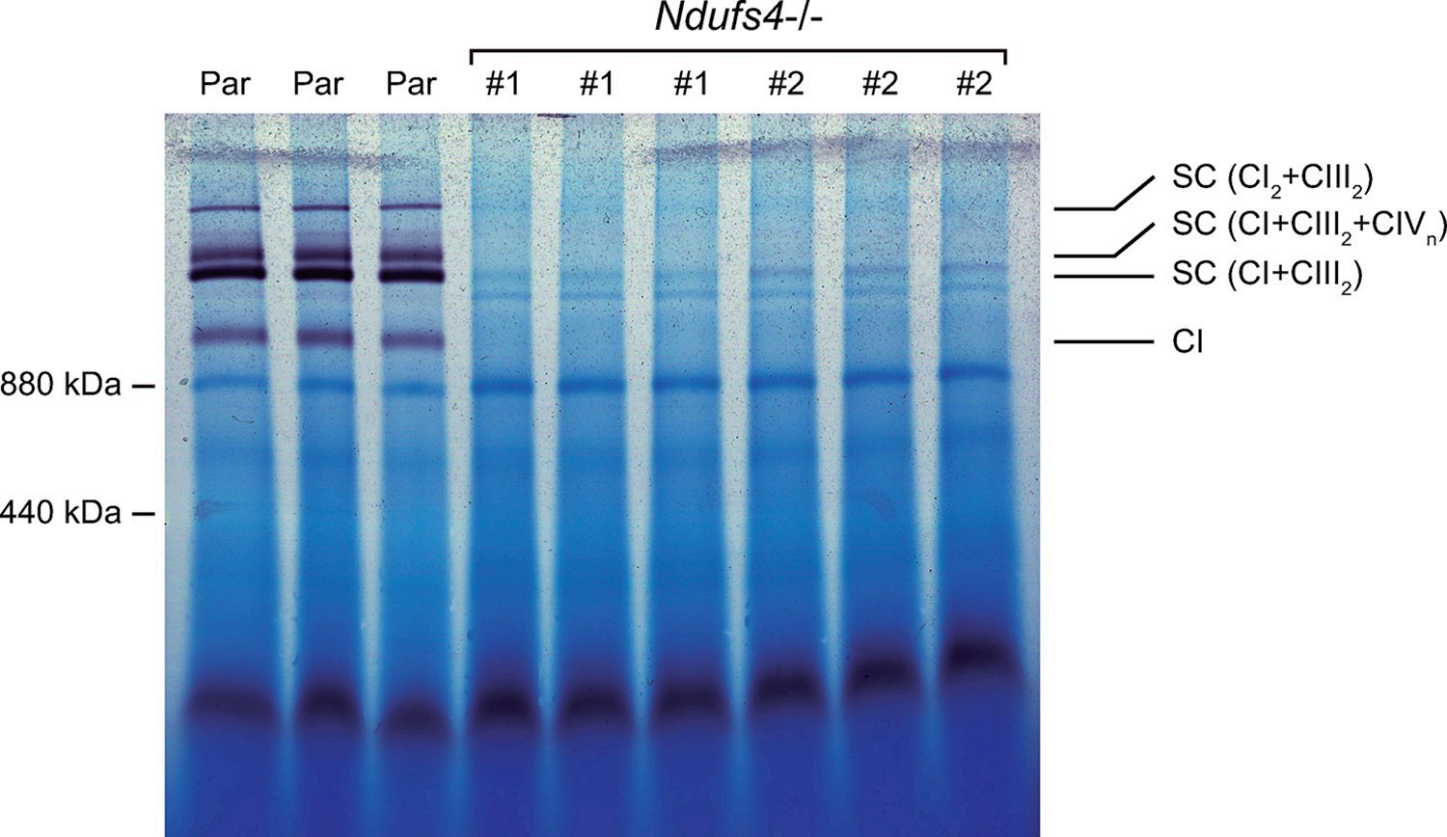
CI activity in *Ndufs4*−/− as assessed by IGA. Native gels were incubated with NADH (as a substrate), and nitro blue tetrazolium (NBT, as the electron acceptor). CI activity is shown in purple.

Finally, CI activity in *Ndufs4*−/− cells was assessed by IGA. Native gels were incubated in a solution containing NADH as a substrate and NBT as the electron acceptor. NADH dehydrogenase activity of CI was displayed by purple bands resulting from in-gel formazan (reduced NBT) precipitation. As shown in Fig 4, free CI and CI-containing SCs were visualized in mitochondrial extracts from parental cells but were undetectable in extracts from *Ndufs4*−/− cells. This result demonstrates that partially assembled CI when Ndufs4 is absent, either on its own or bound with CIII, lacks in-gel enzymatic activity. We address the apparent discrepancy between the CI activity results obtained through IGA and those obtained spectrophotometrically in the discussion section.

### NDUFS4 controls macrophage effector functions

**Fig 5:**
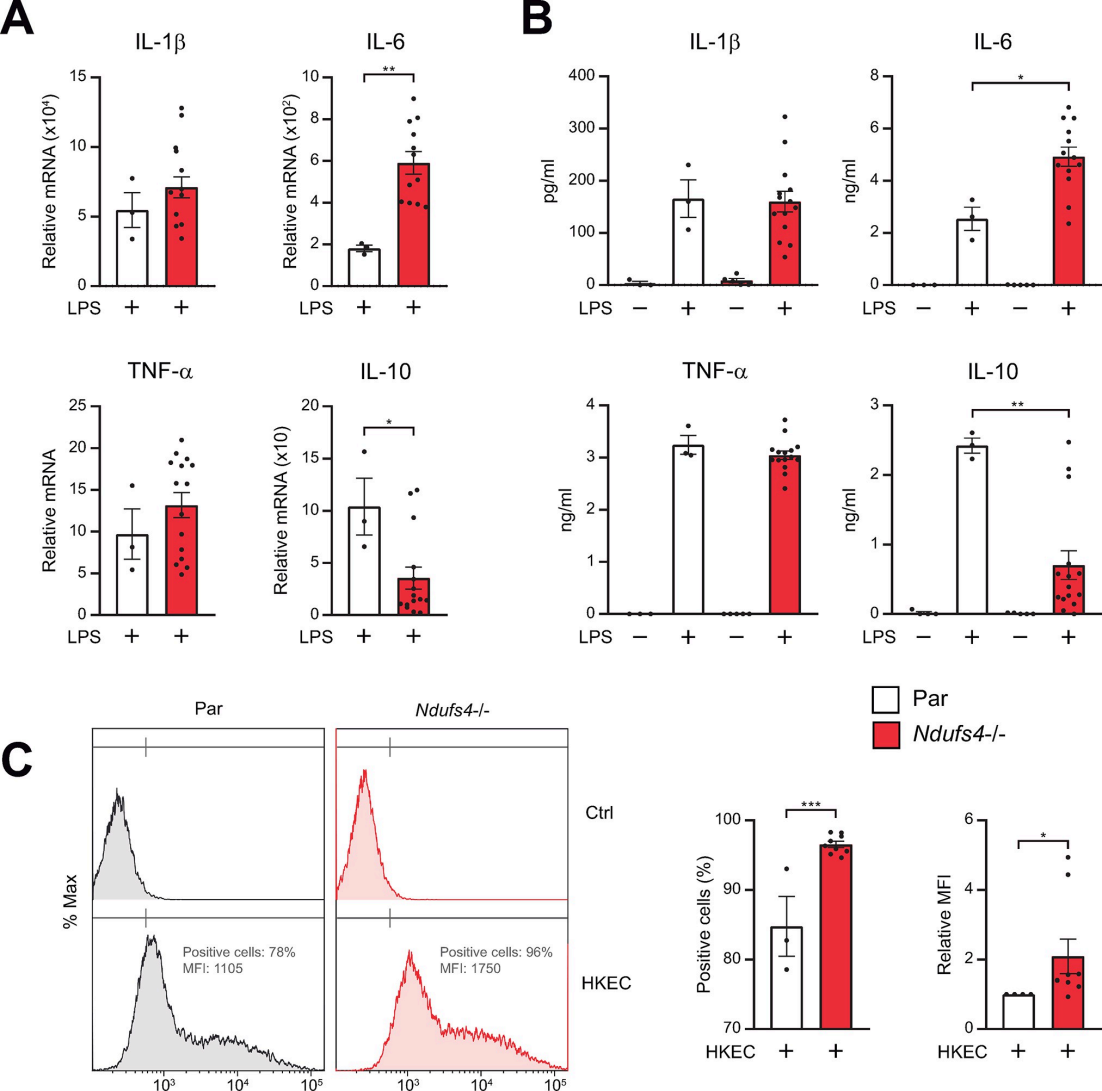
Cytokine profile and phagocytic capacity of *Ndufs4−/−* macrophages. (A, B) Parental (Par), and *Ndufs4−/−* RAW 264.7 cells were left untreated or treated with LPS (100 ng/ml). Supernatants were collected at 8 hours for measurement of cytokine concentrations by ELISA (A). Cells were collected at 4 hours for quantification of cytokine transcripts using real-time PCR (expressed as fold increases versus untreated parental cells) (B). (C) Representative flow cytometry plots (left) showing phagocytosis of FITC labeled heat killed *E*. *coli* (HKEC) and bar graph representing % of fluorescent cells and the MFI values (right). Ctrl, control. Ns, not significant; *, P <0.05; **, P <0.01; ***, P<0.005; ****, P<0.001. Each point represents a biological replicate. In all experiments, 4 to 5 different *Ndufs4−/−* clones were used. Data are shown as the mean ± SEM.

Next, we explored the impact of the absence of NDUFS4 on the cytokine profile and phagocytic capacity of macrophages. The expression of the pro-inflammatory cytokines IL-1β, IL-6 and TNF-α, and the anti-inflammatory cytokine IL-10 in LPS-challenged macrophages was investigated at mRNA and protein levels. As shown in Fig 5A, LPS-activated *Ndufs4*−/− cells expressed higher levels of IL-6 transcripts than parental cells, while the expression of the other pro-inflammatory cytokines was similar in the two cell lines. On the contrary, *Ndufs4*−/− cells expressed lower levels of IL-10 transcripts than parental cells (Fig 5A). The cytokine protein abundance correlated with the corresponding transcript levels. As shown in Fig 5B, secretion of IL-6 by *Ndufs4*−/− macrophages was significantly higher than in controls, whereas their production of IL-10 was deficient. Furthermore, we tested the ability of *Ndufs4*−/− macrophages to phagocyte heat-killed FITC-labeled *E*. *coli*. As shown in Fig 5C, *Ndufs4*−/− showed increased phagocytosis compared to parental cells as measured by mean fluorescence intensity (MFI) and percentage of phagocytic positive cells (FITC+ cells).

## Discussion

In this work we provide new evidence on the key role that NDUFS4 plays in the proper assembly of the CI in macrophages and in controlling their functions. Several studies indicate that NDUFS4 plays a role in the late stage of CI assembly [2, 19, 20] and that the absence of NDUFS4 in patient fibroblasts results in the absence of fully assembled CI, the formation of a partially assembled CI [21–24], a strong decrease of fully assembled respirasomes (SC I + III_2_ + IV_n_) and SC I + III_2_ together with an accumulation of lower molecular-weight SCs [25]. Interestingly, spectrophotometric analysis revealed the presence of some CI activity in NDUFS4 patient samples, despite the absence of fully assembled CI [21, 26–29]. Similarly, all the investigated tissues of the *Ndufs4*−/− mice (pancreas, kidney, liver, lung, brain, heart and muscle) showed absence of fully assembled CI [8, 24] and the large SCs I + III_2_ + IV_n_ were almost absent [8]. In all analyzed tissues of the *Ndufs4*−/− mice, a significant decrease in CI activity was observed, as determined by spectrophotometric assay. However, there were substantial variations in the residual activities among the different knockout tissues, ranging from 9% (in the lung) and 44% (in the heart) [8]. In agreement with the previous studies, our observations confirmed the absence of fully assembled free CI and CI-containing SCs (SC I + III_2_ + IVn, SC I + III_2_) in *Ndufs4*−/− macrophages. Furthermore, we observed a residual CI activity of approximately 50% in *Ndufs4*−/− macrophages when assessed using conventional spectrophotometric methods. However, IGA measurements revealed undetectable CI activity in extracts derived from *Ndufs4*−/− macrophages. The discrepancy in CI activity results obtained by IGA and spectrophotometric assays has been previously documented [8, 25]. The CI spectrophotometric assay measures rotenone-sensitive NADH oxidation. However, the high rate of rotenone-insensitive NADH oxidation in most cell types interferes with the sensitivity of the assay [30]. The NAD+/NADH ratio was measured in *Ndufs4*−/− macrophages (S5 Fig), and a significant decrease of approximately 20% was observed. However, this decrease was modest compared to that observed in other *Ndufs4*−/− tissues, such as the brain [31] and heart [32]. This observation suggests the occurrence of CI-independent NADH oxidation and underscores a major role of *de novo* NAD+ synthesis in maintaining NAD+/NADH homeostasis in macrophages as previously reported [33].

As expected, *Ndufs4*−/− macrophages showed altered OCR both in the absence (Fig 3) and presence of LPS (S6 Fig). Moreover, while the proliferation of *Ndufs4*−/− cells showed no significant changes when cultured in traditional media supplemented with glucose, it was significantly impaired when glucose was substituted with galactose (S7 Fig). Substitution of glucose with galactose in the cell culture medium has been demonstrated to shift ATP production from substrate-level phosphorylation to mitochondrial OXPHOS [34]. Hence, the observed impairment in proliferation in galactose media correlates well with the ETC dysfunction in *Ndufs4*−/− cells. This study also found that *Ndufs4*−/− macrophages had increased basal levels of mitoROS, in line with previous works using other *Ndufs4*−/− cell models [35, 36]. We find this finding puzzling as CI is known to be a primary site of mitoROS production [37] but consistent with oxidative stress stemming from CI dysfunction [38, 39]. Finally, we observed that the MMP was preserved in *Ndufs4*−/− in macrophages, most likely due to the compensatory action of the CIII and CIV proton pumps.

*Ndufs4*−/− macrophage RAW 264.7 cells show increased production of the pro-inflammatory cytokine IL-6 and decreased production of the anti-inflammatory cytokine IL-10 upon LPS-challenge. In line with our results, a previous work shows that palmitic acid, which acts via TLR4 like LPS, induces a higher expression of inflammatory genes in bone marrow derived macrophages from *Ndufs4*−/− mice than those from wild-type mice [35]. The use of the CI inhibitors has generated seemingly opposite results to those obtained using *Ndufs4*−/− models. CI inhibitors metformin and rotenone decreased pro-IL-1β but increased anti-inflammatory IL-10 production in LPS-activated macrophages [40]. Furthermore, a recent study by Xian et al. showed that metformin reduced the severity of LPS- induced pulmonary inflammation in mice by limiting the production of IL-1β and IL-6 [41]. The discrepancy in the results obtained with chemical inhibitors and genetic models may be the result of different degrees of inhibition of CI activity and provide evidence that CI is key in the fine balance between inflammatory and anti-inflammatory responses. It will be interesting to study the effects of the genetic abrogation of different CI subunits on CI activity and their impact on macrophage respiration and effector functions. *Ndufs4*−/− macrophage RAW 264.7 cells also show enhanced phagocytic capacity. This is in accordance with our previous work that showed that rotenone increased the nonopsonic phagocytosis of bacteria [42].

Taken together, our work describes the generation and characterization of a *Ndufs4*−/− murine macrophage cell line. Our data indicates that NDUFS4 is required for the assembly and activity of CI, proper mitochondrial respiration, and ATP production in macrophages. These ETC alterations lead macrophages to a proinflammatory phenotype and to possess an improved phagocytic capacity.

## Supporting information

S1 Table Primer sequences. (DOCX)

S1 Fig CI activity in *Ndufs4−/−* macrophages. The data shown in Fig 2A were reanalyzed and CI activity is shown as CI/CII (left panel) and CI/CIV (right panel). *, P <0.05; **, P <0.01; ***, P<0.005; ****, P<0.001. Each point represents a biological replicate. Data are shown as the mean ± SEM.
(TIF)

S2 Fig mitoROS levels and MMP in *Ndufs4−/−* macrophages. (A) Representative flow cytometry histograms of MitoSOX staining (left) and graph showing relative mitoROS levels (right). (B) Representative flow cytometry histograms (left) of MitoTracker Red CMXRos staining (for MMP) and MitoTracker Green staining (for total mitochondrial mass) and graph showing the ratio of MMP over mitochondrial mass to more accurately determine the potential differences per unit of mitochondrial mass (right). *, P <0.05; **, P <0.01; ***, P<0.005; ****, P<0.001. Each point represents a biological replicate. Data are shown as the mean ± SEM.
(TIF)

S3 Fig Role of Ndufs4 in the proliferation of macrophages. Parental and *Ndufs4−/−* RAW 264.7 cells (1,000) were plated on 96-well plates. The number of viable cells was determined at the indicated time points. Each point represents a biological replicate. Data are shown as the mean ± SD.
(TIF)

S4 Fig Detection of CI subunits in *Ndufs4−/−* macrophages. Left panel, Western blot for NDUFV1 (N module) and NDUFA9 (Q module). ATP5B and Coomassie staining were used as loading controls. Right panel, schematic representation of CI. The N module contains an NADH oxidation site, while the Q module contains a ubiquinone reduction site. P module is involved in proton-pumping activity. The positions of NDUFV1 and NDUFA9 are indicated. IMM, inner mitochondrial membrane; IMS, intermembrane space.
(TIF)

S5 Fig NAD/NADH ratio in *Ndufs4−/−* macrophages. The NAD/NADH ratio was measured through colorimetric detection in deproteinized cell extracts from parental (Par) and *Ndufs4−/−* RAW 264.7 cells. *, P <0.05; **, P <0.01; ***, P<0.005; ****, P<0.001. Each point represents a biological replicate. Data are shown as the mean ± SEM.
(TIF)

S6 Fig Respiration in *Ndufs4−/−* macrophages after exposure to LPS. A representative experiment showing OCR in LPS-pretreated RAW 264.7 sublines before and after the sequential addition of oligomycin (2.6 μM), FCCP (1 μM), and a combination of rotenone (Rot) and antimycin A (AA) (1 μM).
(TIF)

S7 Fig Role of Ndufs4 in the proliferation of macrophages in galactose media. Parental and *Ndufs4−/−* RAW 264.7 cells (40,000) were plated on 6-well plates. The cells were cultured in media containing galactose in the complete absence of glucose. The number of viable cells was determined at the indicated time points. Each point represents a biological replicate. Data are shown as the mean ± SD.
(TIF)

S1 Raw images (PDF)
