# Supplementary material for: Development of a novel in vitro model to study the modulatory role of the respiratory complex I in macrophage effector functions
Source: PLoS One. 2023 Sep 19;18(9):e0291442. doi: 10.1371/journal.pone.0291442 (PMC10508620; doi:10.1371/journal.pone.0291442)
Supplement: S1 Table — (DOCX) [file pone.0291442.s001.docx]

**S1 Table**

**Primers used for cloning**

| CL1 | 5’–CACC**GCGCTGAGACAGGCGATGTTA**−3’ |
| --- | --- |
| CL2 | 5’–AAAC**TAACATCGCCTGTCTCAGCGC**−3’ |
| CL3 | 5’−TAAGTCGAC**TCGCTGAGACAGGCGATGTTAGG**TAGATAGTTAGCGACAATCCCGGGTCTCCGCG−3’ |
| CL4 | 5’−ATTGGAT**CCTAACATCGCCTGTCTCAGCGA**GGCTGCAGGAATTCGATCTGGG−3’ |

Bold: target sequences, PAM sequences are underlined. Red: restriction enzyme sites (SalI in CL5 and CL7, BamHI in CL6 and CL8), BbsI compatible overhangs are underlined. Orange: stop sequences. Green: sequences that match with pBluescript II KS+.

**Primers used for Real time PCR**

| RT1 (Hprt-FWD) | 5’−GCGTCGTGATTAGCGATGATG−3’ |
| --- | --- |
| RT2 (Hprt-REV) | 5’−GAGCAAGTCTTTCAGTCCTGT−3’ |
| RT3 (IL-1β-FWD) | 5’−TGTCCTCATCCTGGAAGGTC−3’ |
| RT4 (IL-1β-REV) | 5’−TGTGAAATGCCACCTTTTGA−3’ |
| RT5 (IL-6-FWD) | 5’−CTTCCATCCAGTTGCCTTCT−3’ |
| RT6 (IL-6-REV) | 5’−CTCCGACTTGTGAAGTGGTATAG−3’ |
| RT7 (IL-10-FWD) | 5’−ACAGCCGGGAAGACAATAAC−3’ |
| RT8 (IL-10-REV) | 5’−CAGCTGGTCCTTTGTTTGAAAG−3’ |
| RT9 (Tnf-α-FWD) | 5’−AGCCGATGGGTTGTACCTTGTCTA−3’ |
| RT10 (Tnf-α-REV) | 5’−TGAGATAGCAAATCGGCTGACGGT−3’ |
