# Supplementary figures and images for: Development of a novel in vitro model to study the modulatory role of the respiratory complex I in macrophage effector functions
Source: PLoS One. 2023 Sep 19;18(9):e0291442. doi: 10.1371/journal.pone.0291442 (PMC10508620; doi:10.1371/journal.pone.0291442)

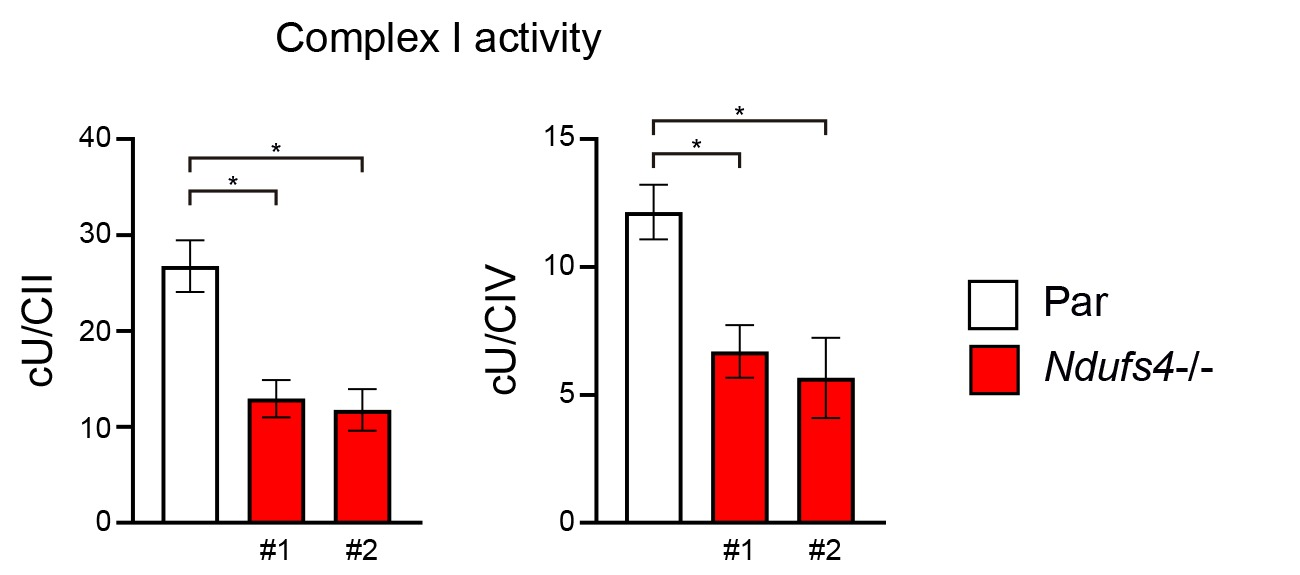

Supplement: S1 Fig — The data shown in Fig 2A were reanalyzed and CI activity is shown as CI/CII (left panel) and CI/CIV (right panel). *, P <0.05; **, P <0.01; ***, P<0.005; ****, P<0.001. Each point represents a biological replicate. Data are shown as the mean ± SEM. (TIF) [file pone.0291442.s002.tif]

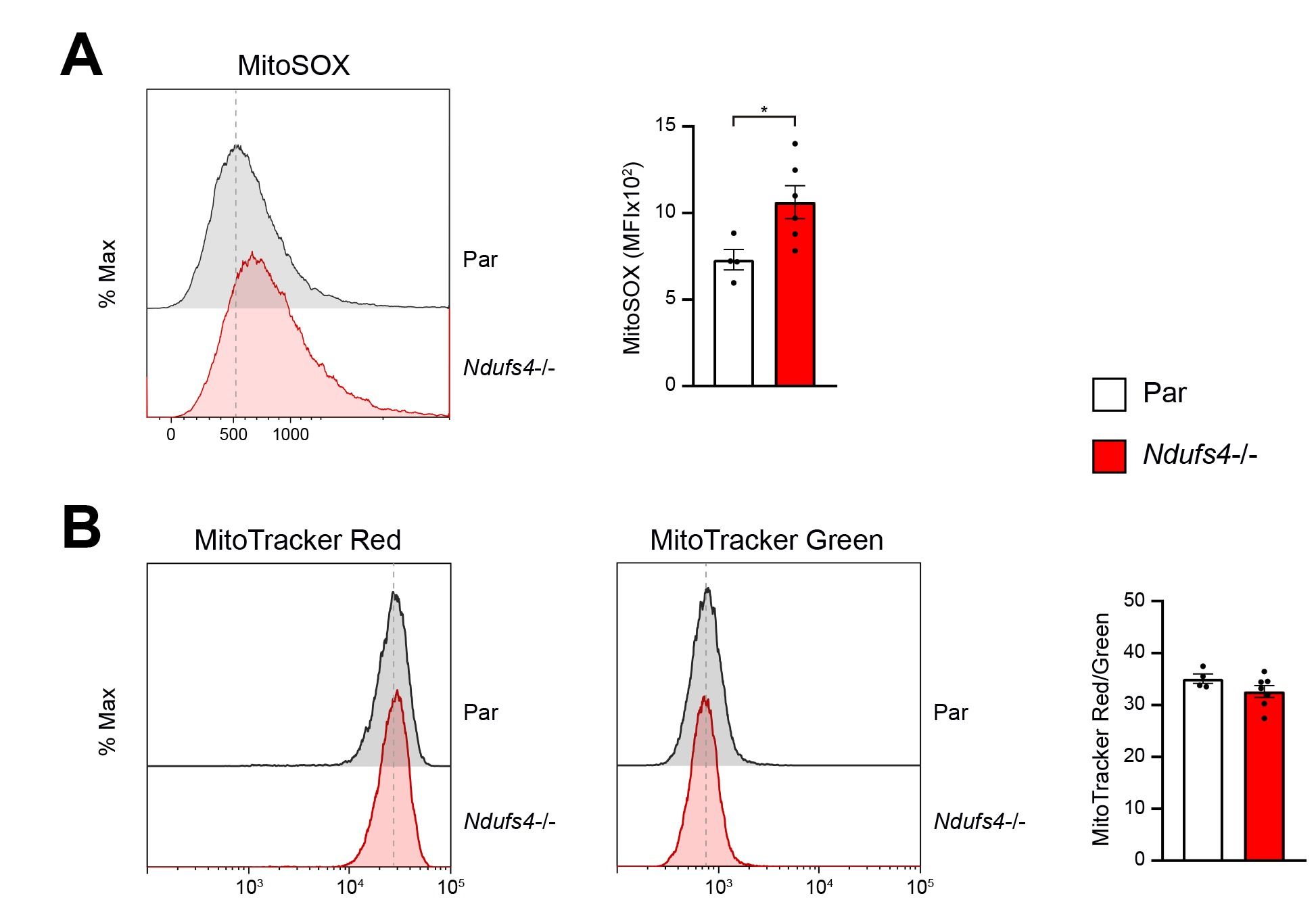

Supplement: S2 Fig — (A) Representative flow cytometry histograms of MitoSOX staining (left) and graph showing relative mitoROS levels (right). (B) Representative flow cytometry histograms (left) of MitoTracker Red CMXRos staining (for MMP) and MitoTracker Green staining (for total mitochondrial mass) and graph showing the ratio of MMP over mitochondrial mass to more accurately determine the potential differences per unit of mitochondrial mass (right). *, P <0.05; **, P <0.01; ***, P<0.005; ****, P<0.001. Each point represents a biological replicate. Data are shown as the mean ± SEM. (TIF) [file pone.0291442.s003.tif]

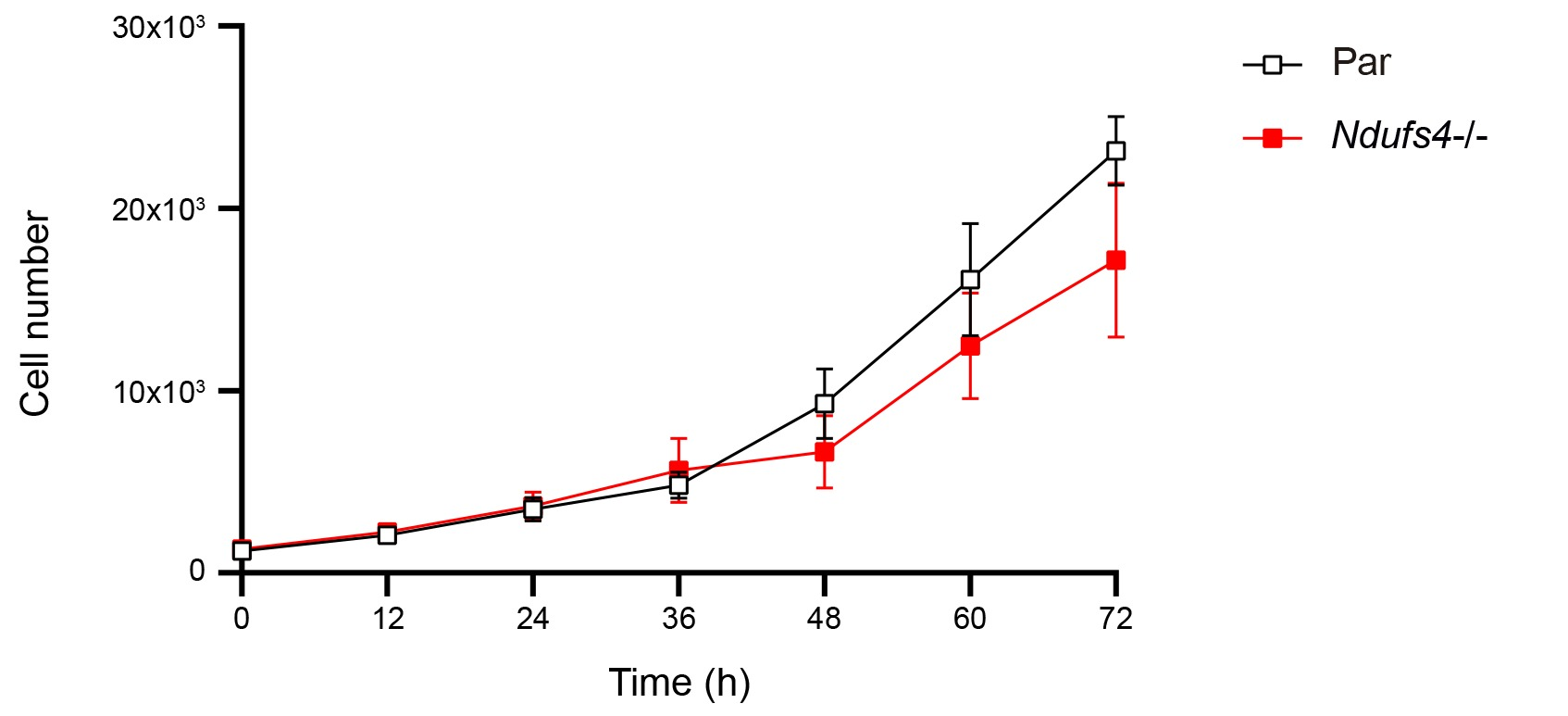

Supplement: S3 Fig — Parental and Ndufs4−/− RAW 264.7 cells (1,000) were plated on 96-well plates. The number of viable cells was determined at the indicated time points. Each point represents a biological replicate. Data are shown as the mean ± SD. (TIF) [file pone.0291442.s004.tif]

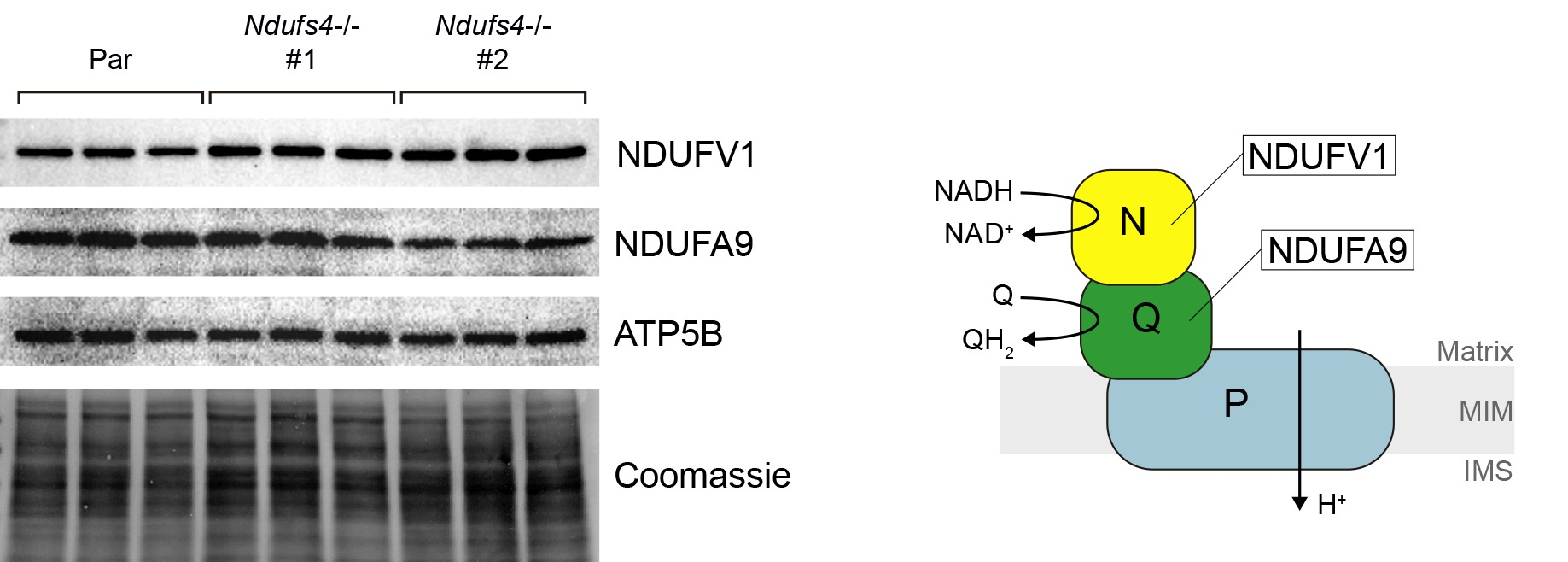

Supplement: S4 Fig — Left panel, Western blot for NDUFV1 (N module) and NDUFA9 (Q module). ATP5B and Coomassie staining were used as loading controls. Right panel, schematic representation of CI. The N module contains an NADH oxidation site, while the Q module contains a ubiquinone reduction site. P module is involved in proton-pumping activity. The positions of NDUFV1 and NDUFA9 are indicated. IMM, inner mitochondrial membrane; IMS, intermembrane space. (TIF) [file pone.0291442.s005.tif]

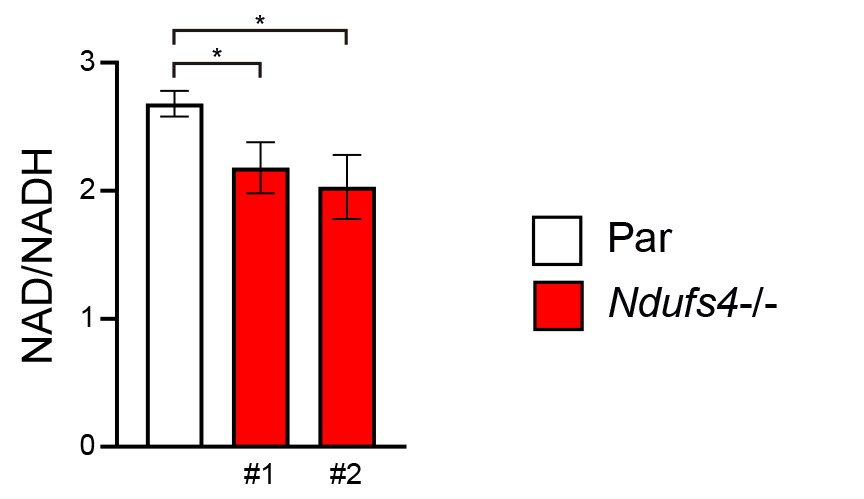

Supplement: S5 Fig — The NAD/NADH ratio was measured through colorimetric detection in deproteinized cell extracts from parental (Par) and Ndufs4−/− RAW 264.7 cells. *, P <0.05; **, P <0.01; ***, P<0.005; ****, P<0.001. Each point represents a biological replicate. Data are shown as the mean ± SEM. (TIF) [file pone.0291442.s006.tif]

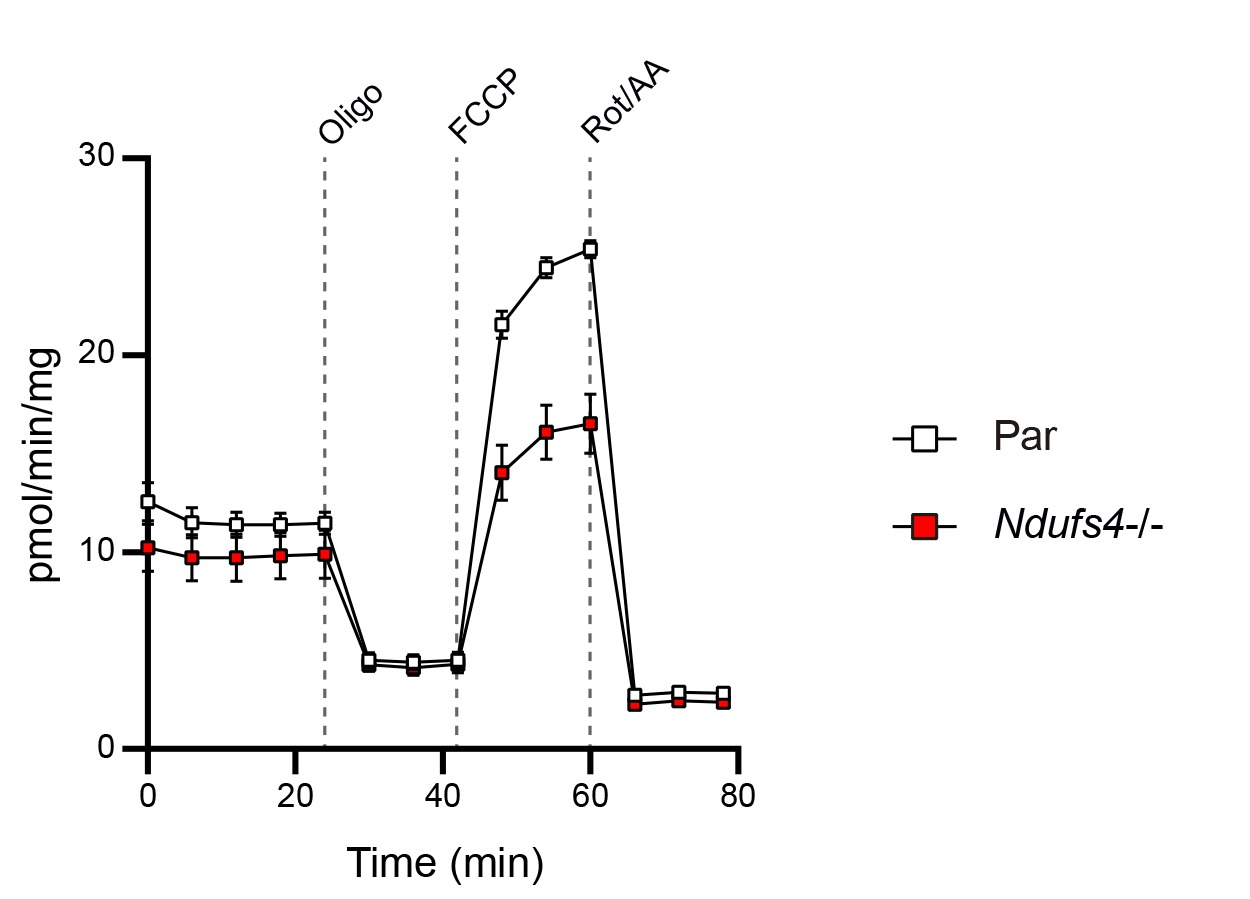

Supplement: S6 Fig — A representative experiment showing OCR in LPS-pretreated RAW 264.7 sublines before and after the sequential addition of oligomycin (2.6 μM), FCCP (1 μM), and a combination of rotenone (Rot) and antimycin A (AA) (1 μM). (TIF) [file pone.0291442.s007.tif]

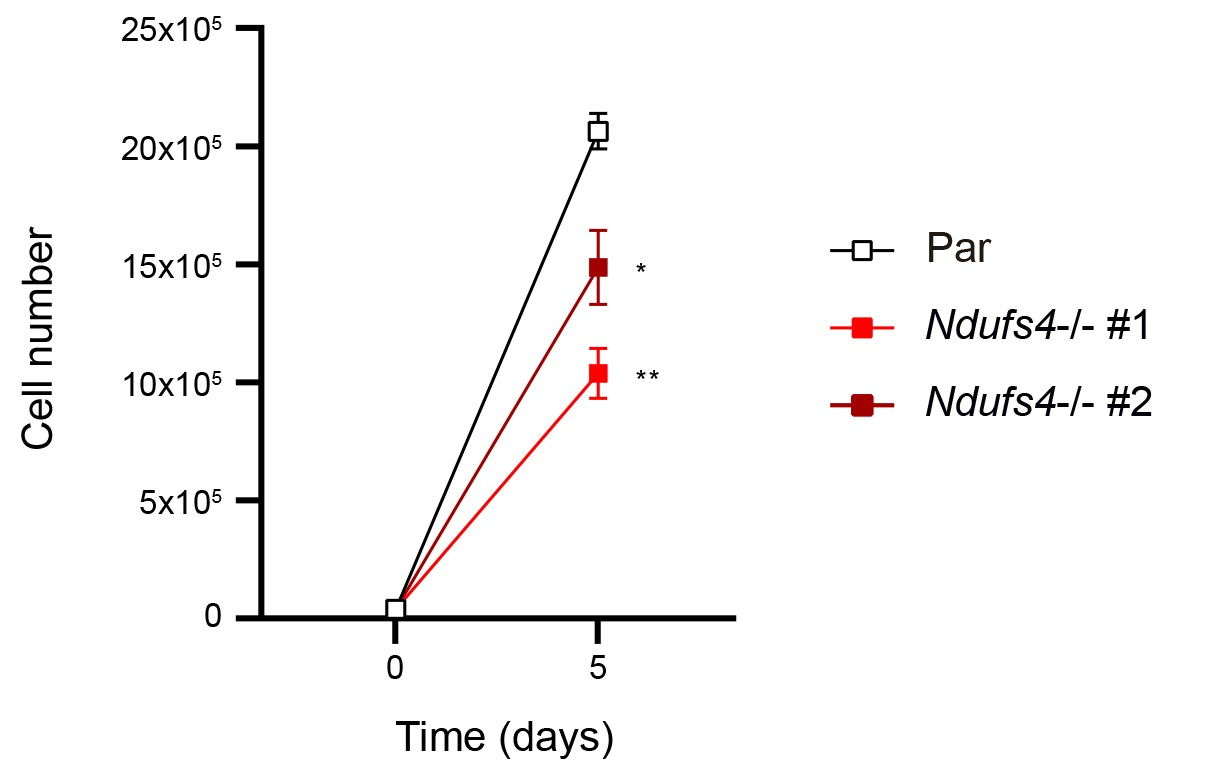

Supplement: S7 Fig — Parental and Ndufs4−/− RAW 264.7 cells (40,000) were plated on 6-well plates. The cells were cultured in media containing galactose in the complete absence of glucose. The number of viable cells was determined at the indicated time points. Each point represents a biological replicate. Data are shown as the mean ± SD. (TIF) [file pone.0291442.s008.tif]

**Fig 1**

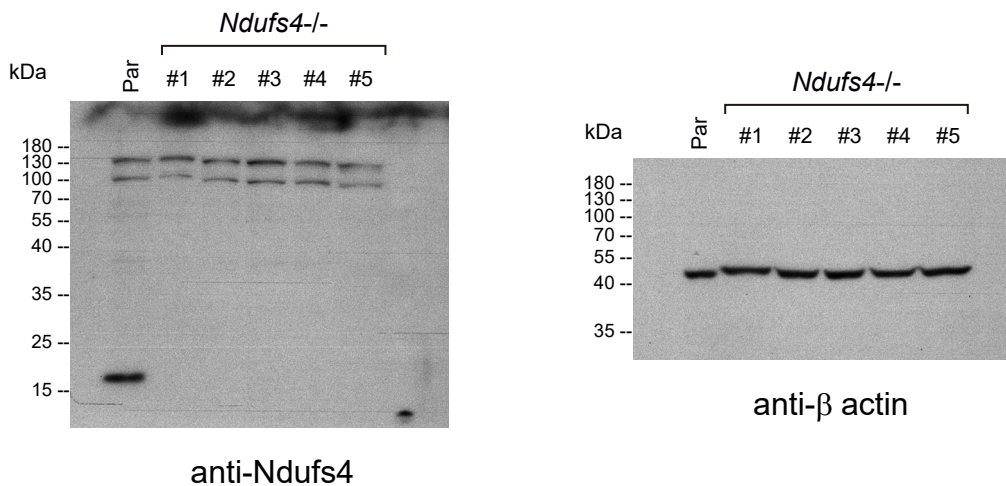

**Fig 3**

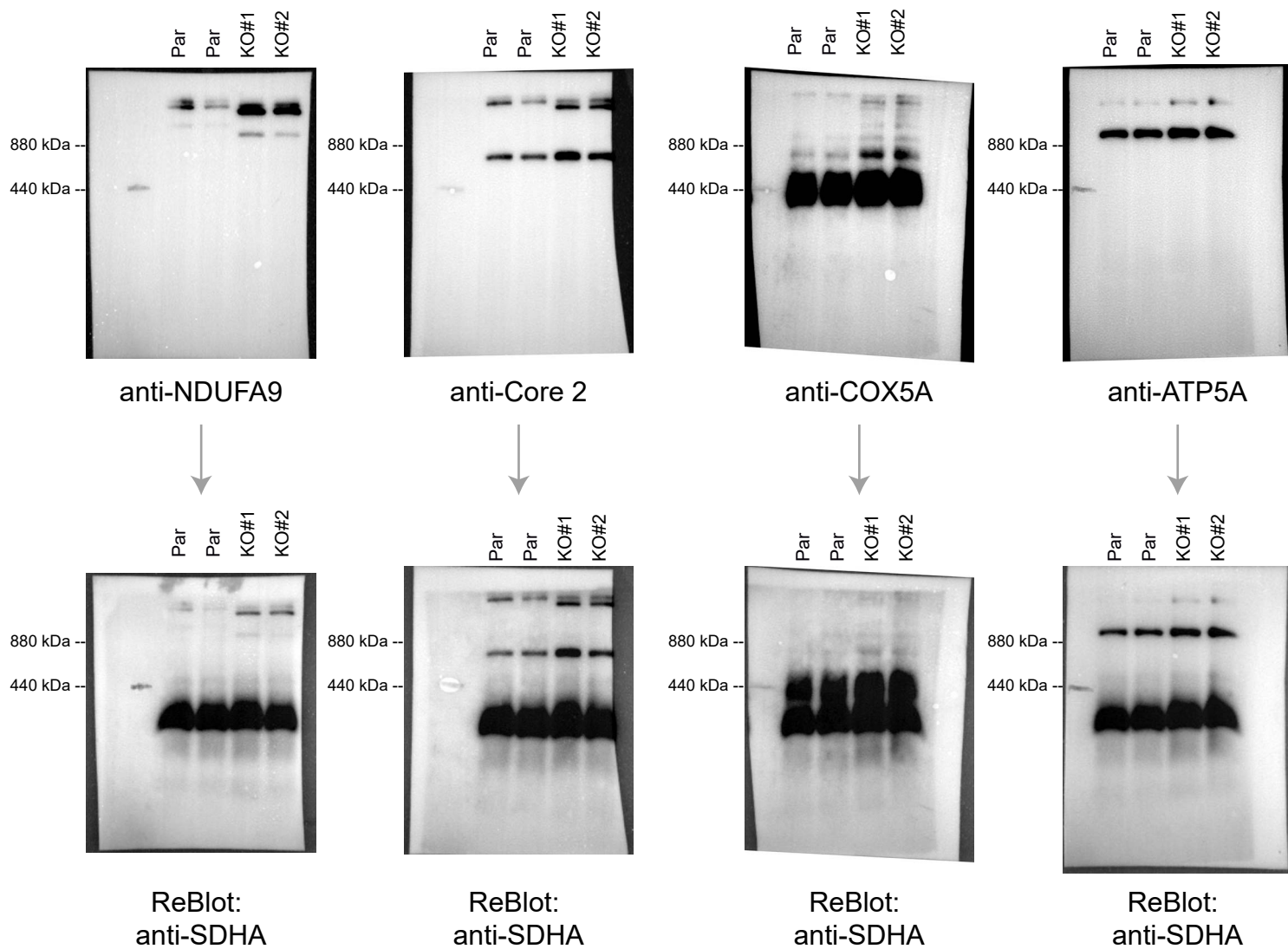

**Fig 4**

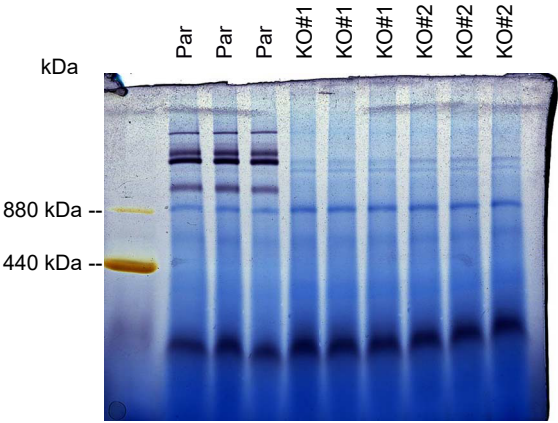

**S4 Fig**

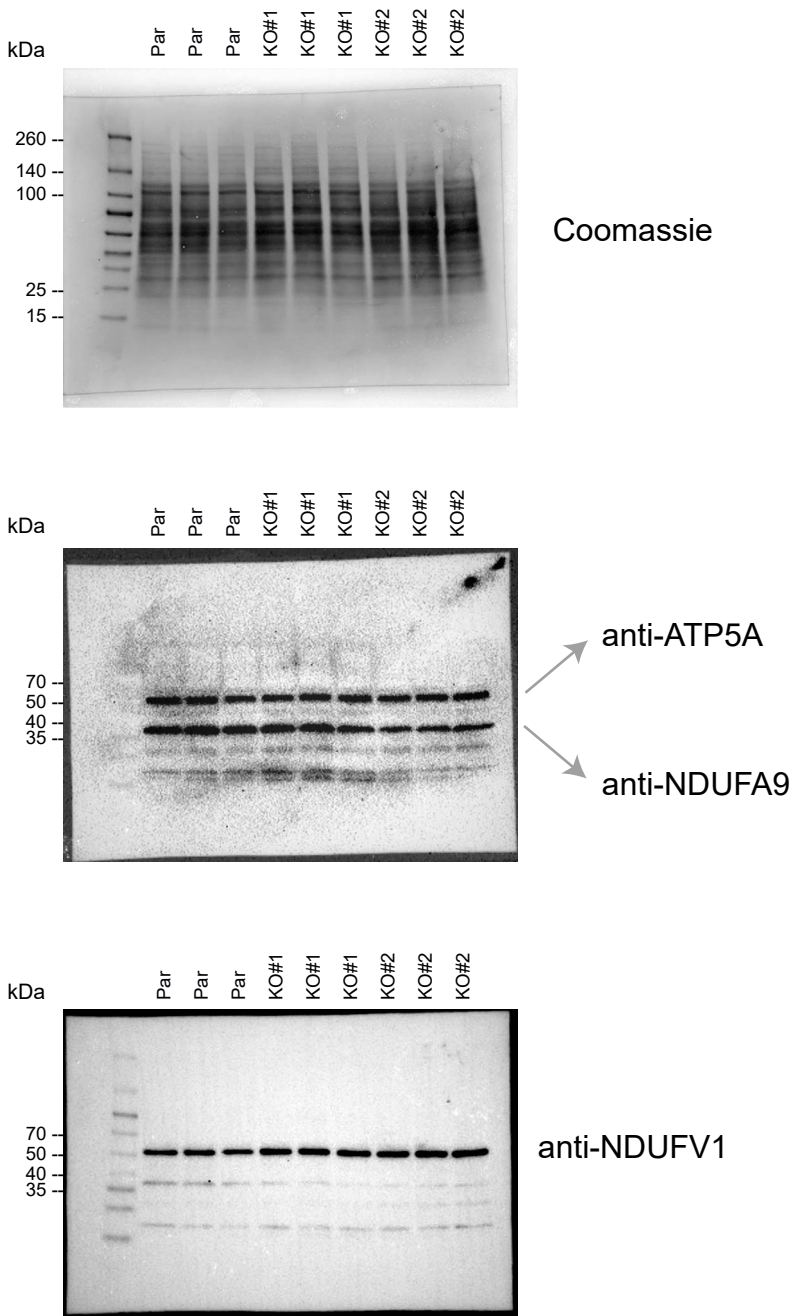

Supplement: S1 Raw images — (PDF) [file pone.0291442.s009.pdf]
